# Comparison of CT-guided thoracic sympathetic nerve block and radiofrequency in the treatment of primary palmar hyperhidrosis

**DOI:** 10.3389/fsurg.2023.1126596

**Published:** 2023-05-31

**Authors:** Li Zhang, Shuang-shuang Xu, Xiao-lan Liu, Wei Zhao, Ying Ma, Bing Huang

**Affiliations:** ^1^ The Department of Anesthesiology and Pain Research Center, the First Affiliated Hospital of Jiaxing University, Jiaxing, China; ^2^ Graduate School, Bengbu Medical College, Bengbuy, China; ^3^ Graduate School, Zhejiang Chinese Medical University, Hangzhou, China

**Keywords:** hyperhidrosis, palmar, sympathetic, nerve block, autonomic nerve block, radiofrequency therapy

## Abstract

**Background:**

Primary palmar hyperhidrosis (PPH) is a condition marked by an overactive secretion of the hand's exocrine glands and is frequently hereditary. The profuse sweating associated with this condition can significantly impair the patient's daily activities and quality of life.

**Objective:**

The objective of this study was to compared the benefits and drawbacks of thoracic sympathetic block and thoracic sympathetic radiofrequency in the treatment of PPH.

**Methods:**

A retrospective analysis was conducted on 69 patients. They were divided into groups A and B according to their treatment. Group A (34 cases) received CT-guided percutaneous thoracic sympathetic nerve chain anhydrous alcohol chemical damage block, and group B (35 cases) received CT-guided percutaneous thoracic sympathetic nerve chain radiofrequency thermocoagulation.

**Results:**

Palmar sweating disappeared immediately after the operation. The recurrence rates at 1, 3, 6, 12, 24, and 36 months were 5.88% vs. 2.86% (*P* > 0.05), 20.59% vs. 5.71% (*P* > 0.05), 32.35% vs. 11.43% (*P* < 0.05),32.35% vs. 11.43% (*P* < 0.05), 25% vs. 14.71% (*P* < 0.05), and 68.75% vs. 20.59% (*P* < 0.05), respectively. The incidence of intercostal neuralgia and compensatory hyperhidrosis was higher in group A compared with of group B (52.94% vs. 22.86%, *P* < 0.05; 55.88% vs. 22.86%, *P* < 0.05).

**Conclusion:**

Both methods were found to be effective in treating PPH, but thoracic sympathetic radiofrequency had a longer-term effect, a lower recurrence rate, and a lower incidence of intercostal neuralgia and compensatory hyperhidrosis than a thoracic sympathetic block.

## 1. Introduction

Primary palmar hyperhidrosis (PPH) is a condition characterized by excessive secretion of the exocrine glands in the hand, which is often accompanied by hyperhidrosis in other areas of the body, including the head, face, armpit, or foot (1). In China, the disease rate in China is estimated to be 2.08%, with 25.40% of patients having a family history of PPH (2). Despite extensive research, the pathogenesis of PPH remains unknown (3). Thoracoscopic sympathectomy (ETS), is considered the most effective treatment for PPH (4), but its use is limited due to a high incidence of compensatory hyperhidrosis (5). We have previously employed CT-guided thoracic sympathetic chain anhydrous alcohol chemical damage block to treat palmar hyperhidrosis (6–8). However, this treatment has a limited duration, and anhydrous alcohol can spread to the intercostal nerve, leading to intercostal neuralgia and a high incidence of compensatory hyperhidrosis. Therefore, we introduced CT-guided radiofrequency thermocoagulation (9) in the treatment of PPH. The aim of this study was to assess the pros and cons of two novel technologies for managing PPH.

## 2. Material and methods

### 2.1. General information

This study was approved by the ethics committee of the First Hospital of Jiaxing, the Affiliated Hospital of Jiaxing University (Approval number: ls2018-141). From January 2017 to December 2018, we collected data from patients who underwent surgery for PPH at our hospital. The patients were divided into two groups, A and B based on the surgical procedures they received. Group A (*n* = 34) received a CT-guided percutaneous thoracic sympathetic nerve block with anhydrous alcohol, while group B (*n* = 35) received CT-guided percutaneous radiofrequency thermocoagulation to physically damage the thoracic sympathetic nerve chain.

### 2.2. Patient Criteria

**Inclusion criteria**:

(1) Patients who met the diagnostic criteria for primary hyperhidrosis established by the multidisciplinary working group of the American Academy of Dermatology (10, 11);
(2) Patients between the ages of 18 and 45;
(3) Patients who had not undergone previous surgical treatment;
(4) Patients with a Hyperhidrosis Disease Severity Scale (HDSS) (3, 12) scored 3–4.

**Exclusion criteria**:

(1) Patients who were allergic to alcohol or local anesthetics;
(2) Pregnancy or preparing for pregnancy, patients with severe thoracic deformity, lung or puncture site infection, or other serious systemic diseases that would preclude the operation;
(3) Patients with secondary hyperhidrosis.

### 2.3. Operation method

Prior to the operation, patients were informed about the operation process and risks and were required to sign an informed consent form. Patients were advised to refrain from consuming any food or drink for at least 8 h before the operation. A trocar was inserted in the upper limb vein for later use. The patient was then sent to the CT operating room, and asked to lie prone on the CT console and position their hands close to the outside of their body with palms up. Vital signs, palm temperature (T), and pulse index (PI) were monitored throughout the operation.

**Figure 1:**
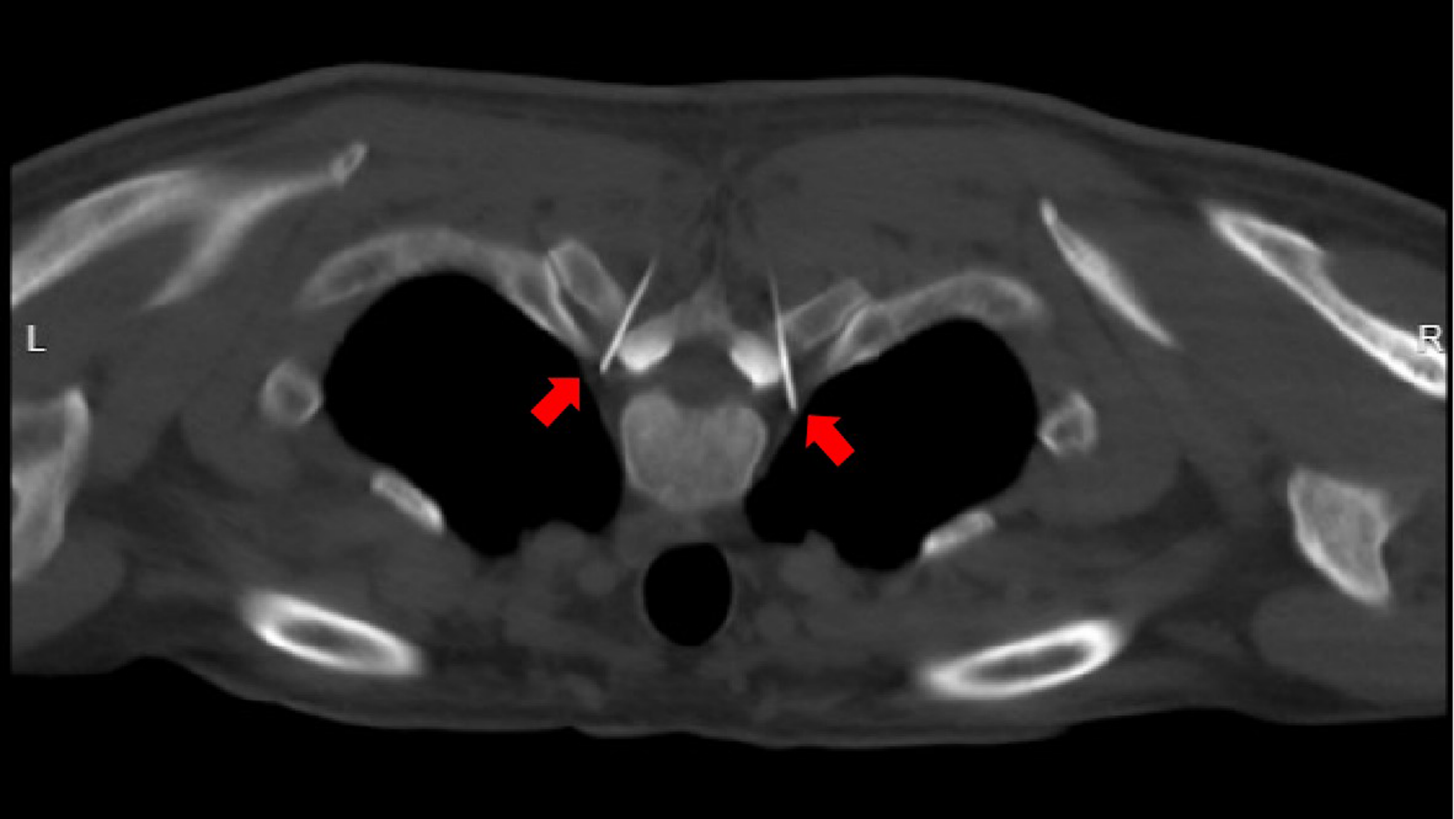
Under CT guidance, the needle tip reaches the predetermined target point (indicated by the red arrow) along the designed puncture path.

**Figure 2:**
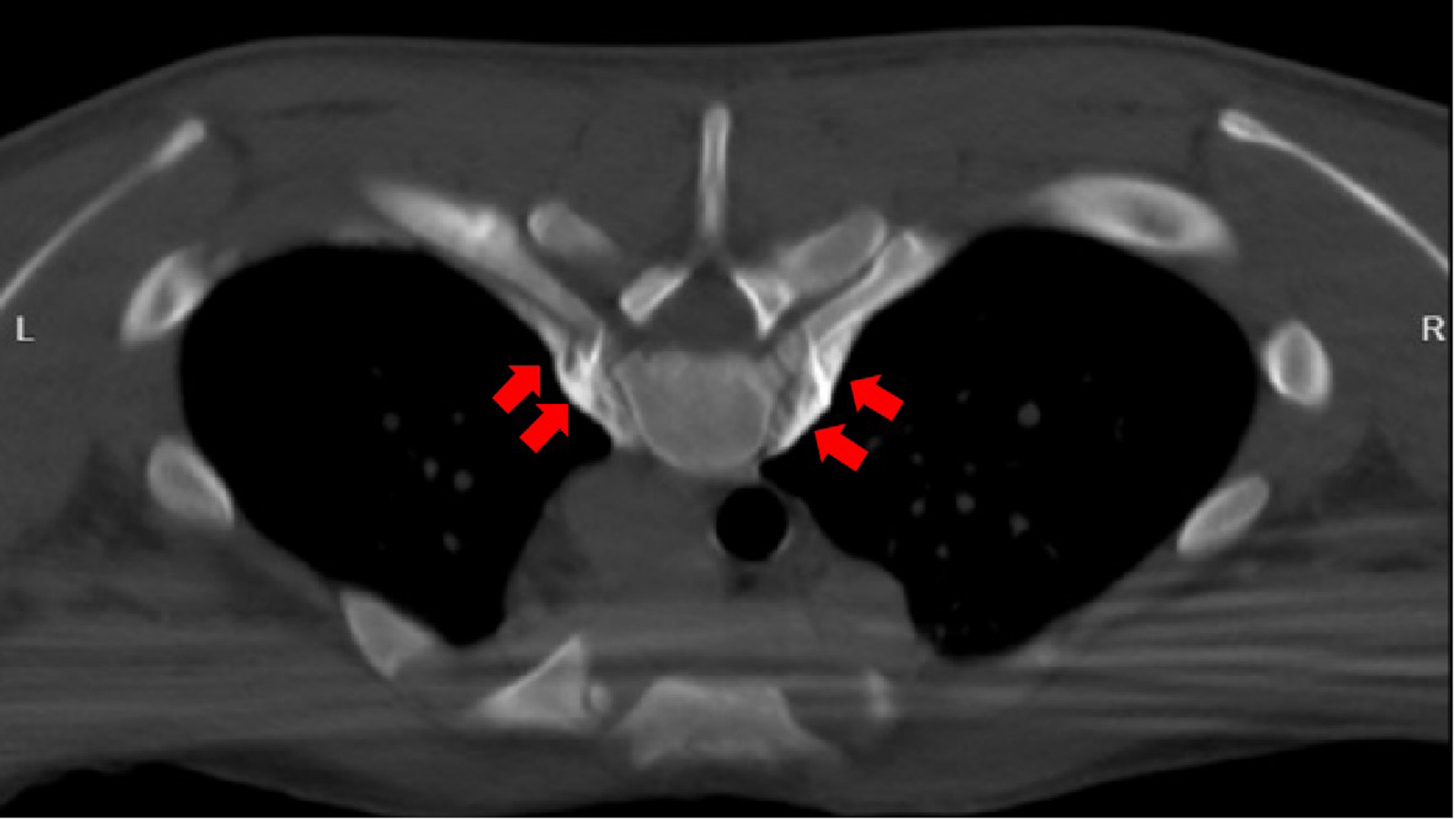
Successful puncture with lidocaine test. 2.5 ml of absolute alcohol (each 1 ml contains 0.9 ml of absolute alcohol and 0.1 ml of 30% iohexol) is injected on both sides, as shown by the red arrow in the figure.

**Figure 3:**
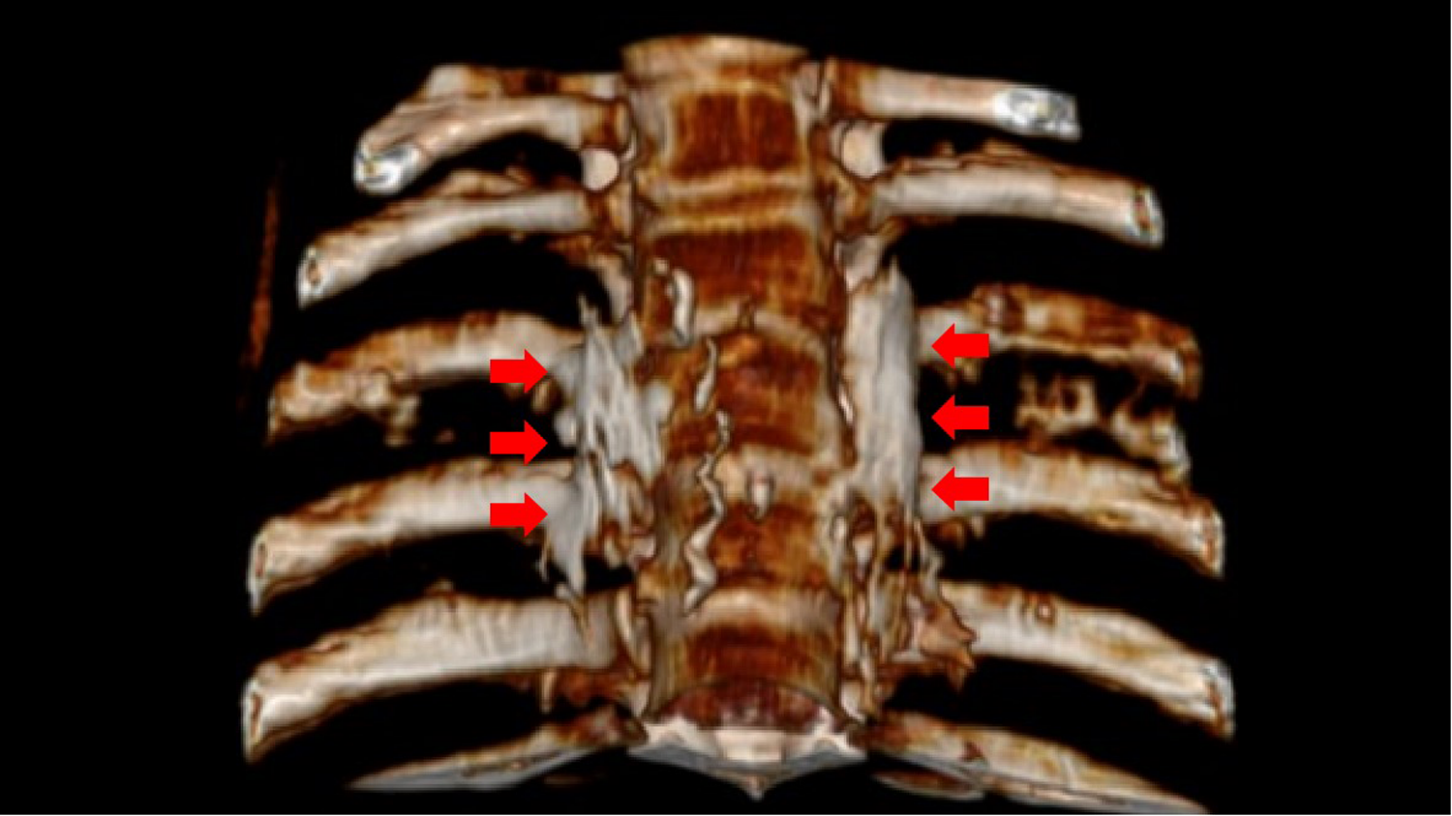
The distribution of absolute alcohol on both sides after three-dimensional CT reconstruction, which wraps around the small head of the 4th rib and the lateral edge of the 4th vertebral body, as indicated by the red arrow in the figure.

All patients underwent treatment at the sympathetic nerve chain at the level of the 4th thoracic vertebra level (T4). The CT scan frame was set on the chest CT localization image, covering the 1st to 4th ribs. Subsequently, an axial scan with a layer thickness of 3 mm was conducted to determine the layer encompassing the upper border of the small head of the fourth rib, which was identified as the optimal puncture site. A puncture path was then charted based on this layer. The process for each group is described below:

Group A (6–8): Puncture target points on both sides were pulled straight to the back skin through the lamina-transverse process gap, and the intersection point with the skin was identified as the puncture entry point. The puncture depth and angle were measured using the CT software ruler. Following the administration of local anesthesia, a No. 7 puncture needle with a length of 10 cm was used to puncture the target point according to the designed puncture path (Figure 1). The needle core was then removed, and 3 ml of 1% lidocaine (0.1 ml of 30% iohexol contrast agent for development) was injected on each side. CT scanning was then repeated. If the injected drug solution wrapped around the small head of the fourth rib and the lateral edge of the fourth vertebral body without entering the spinal canal along the intervertebral foramen (Figure 2), and T and PI were observed to increase significantly, 2.5 ml of absolute alcohol (with iohexol) was slowly injected into each side after 20 min. CT scanning was repeated, and a three-dimensional reconstruction of the image was performed (Figure 3). The distribution of the injected liquid and complications such as pneumothorax and bleeding were then observed. Finally, the needle was pulled out, and the puncture point was covered with a sterile dressing.

**Figure 4:**
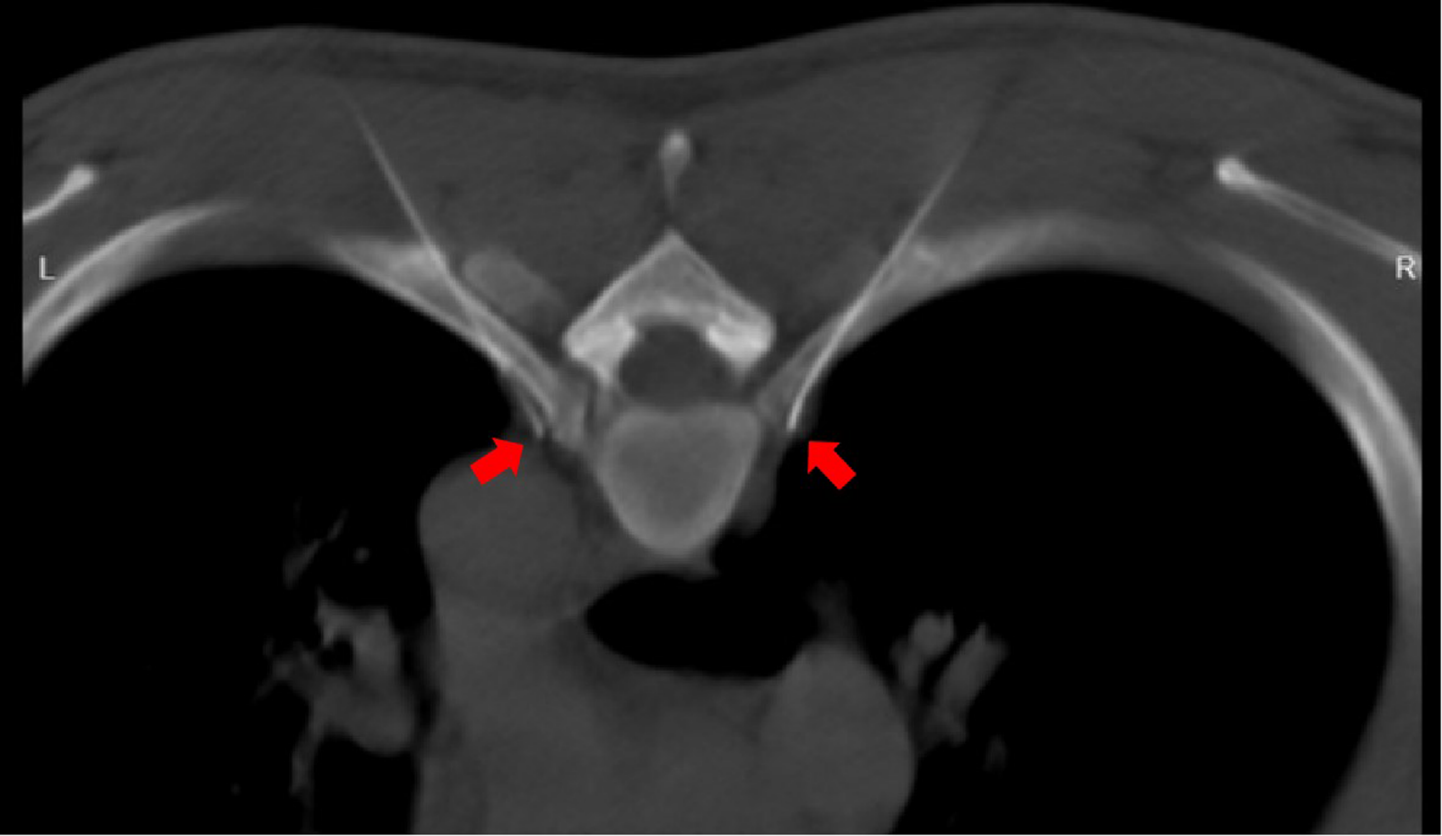
CT-guided puncture reaching the predetermined target point along the designed path (indicated by the red arrow).

**Figure 5:**
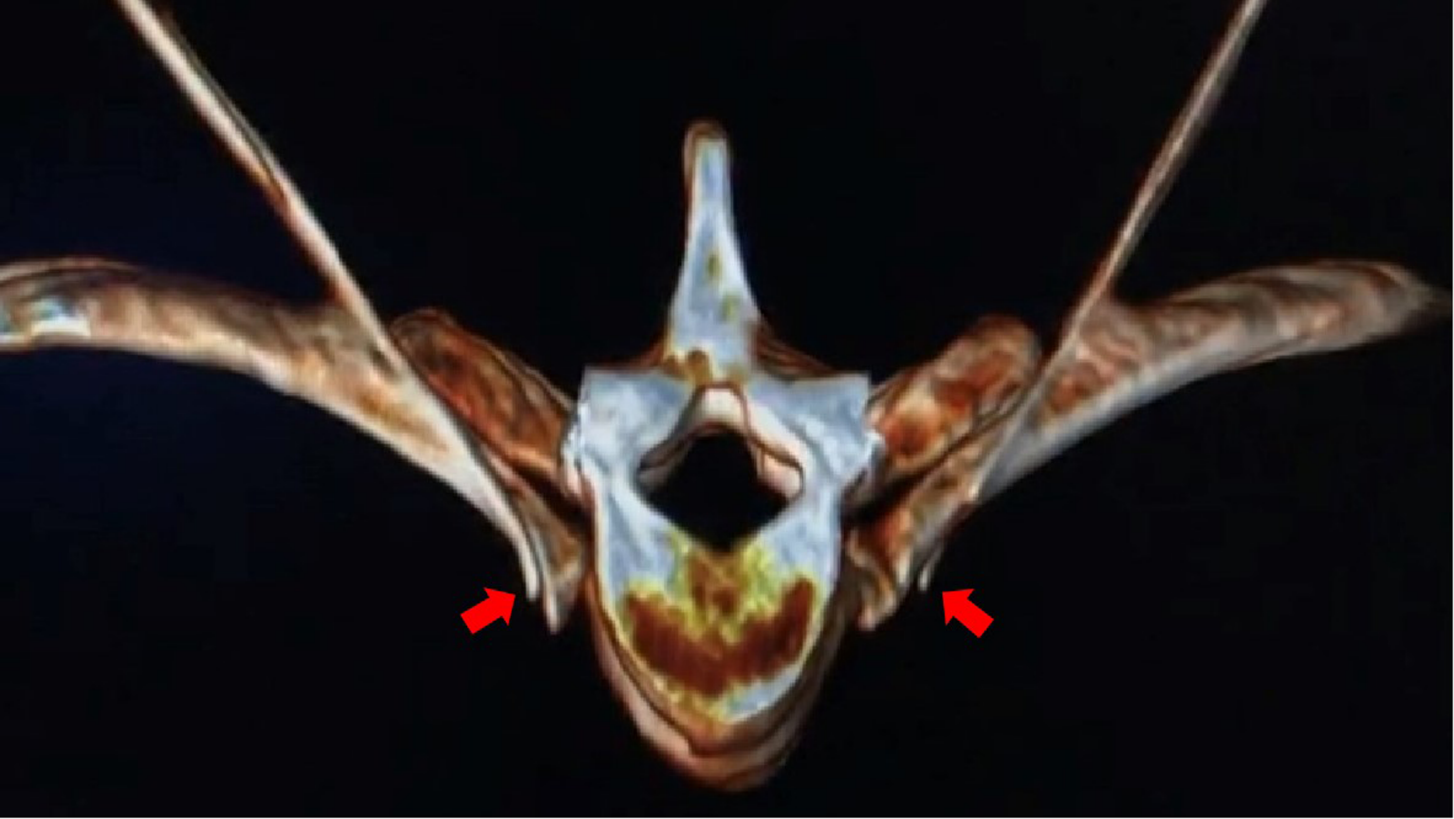
After the three-dimensional reconstruction of CT, the needle tips on both sides reach the anterior superior edge of the 4th rib head and the lateral wall of the T4 vertebral body. (indicated by the red arrow).

Group B (9): Puncture paths were designed as in Group A. Following local anesthesia, a 10 cm No.7 blunt thoracic sympathetic RF puncture needle (with a bare end of 10 mm) was used to puncture the target point until the needle tip was close to the front upper edge of the 4th rib small head (Figure 4). A three-dimensional reconstruction of the CT image was carried out to confirm the position of the puncture tip (Figure 5). The RF needle core was removed, and the RF electrode was inserted to perform electrophysiological tests on motor and sensory nerves, respectively. RF thermocoagulation was conducted at 95°C for 300 s if a current below 1.5 mA elicited no numbness or muscle twitching within the spinal nerve innervation area. T and PI were monitored before and after the procedure, after which the treatment was terminated.

### 2.4. Observation and follow-up

To evaluate the relief of hyperhidrosis in the hands of patients after the operation, observed the changes in palm temperature (T), perfusion index (PI), finger pulse oxygen saturation (SPO_2_), and heart rate (HR) before and after treatment, as well as any occurrences of local hematoma, spinal cord injury, vascular embolism, pneumothorax, Horner's syndrome, and other complications. We conducted telephone follow-ups on the 1st, 3rd, 6th, 12th, 24th, and 36th months after the operation. We evaluated the incidence of unrelated nerve injury (intercostal neuralgia), compensatory sweating (13), and the recurrence rate of hand sweating.

The degree of postoperative hyperhidrosis was scored 1–2 points based on the severity of the symptoms and the HDSS score scale, indicating a satisfactory curative effect. A postoperative recurrence was considered if one or both hands perspired again after the operation, and the HDSS score reached 3–4 points. We subsequently recorded the recurrence time.

### 2.5. Statistical analysis

Data were analyzed using SPSS 22.0 software. Measurement data were expressed as mean standard deviation (x ± s). The *t*-test was used to make a comparison between the two groups, and the rank sum test to compare the skewness distribution measurement data between the two groups. The rank data of different groups was expressed as a percentage and compared the average rank values between groups using the Mann-Whitney *U* test. Data were compared between the two groups using the *χ*^2^ test (Chi-squared test). PI, T, and SpO_2_ were corrected by repeated measurement ANOVA and the Greenhouse-Geisser method. The inspection level was set at *P* < 0.05.

## 3. Results

### 3.1. General demographic information

Analysis of the revealed that age, sex, and BMI were not significantly different between the two groups (*P* > 0.05) (Table 1).

**Table 1:** Comparison of the general conditions between two groups of patients.

Group	Age	BMI	Gender (*n*/%)	
	Year (P25,P75)	(Kg/m^2^)	Male	Female
A	25 (20.0,30.5)	20.7 ± 2.6	15/44.1	19/55.9
B	23 (20.0,28.0)	21.0 ± 3.0	17/48.6	18/51.4
Z/t/*x*^2^ value	Z = −1.220	t = −0.388	*x*^2 ^= 0.138	
*P* value	0.222	0.699	0.711	

The study involved 69 patients (138 sides), all of whom underwent either chemical damage to the sympathetic nerve chain or radiofrequency thermocoagulation with successful outcomes. Immediately following the procedure, all patients' hands exhibited dryness, and no significant complications such as hematoma, vascular embolism, or paraplegia were observed. During the operation, three patients experienced a decrease in blood pressure and HR, but they immediately recovered after using an atropine needle.

In group A, CT scans during the operation revealed unilateral drug infiltration into the cervical sympathetic ganglia at the head in four cases, and a slight Horner's syndrome was observed. However, the syndrome disappeared within 30 min after injecting 5 ml of normal saline into the stellate ganglion (14). In group B, CT scans after a radiofrequency operation revealed a small amount of pneumothorax in one patient who had no complaints. The pneumothorax did not worsen after the patient stayed in bed for 24 h and took oxygen.

### 3.2. Monitoring indicators before and after the operation

There was no significant difference in HR and SpO_2_ between the two groups before and after the operation (*P* > 0.05). After the operation, both groups showed a significantly higher palm T in both hands significantly increased compared to before the operation, with a statistically significant difference (*P* < 0.05). Additionally, both groups' peripheral PI of both hands significantly increased after surgery compared to before surgery (*P* < 0.05) (Tables 2–4).

**Table 2:** Comparison of preoperative and postoperative HR and SPO2 between the two groups.

	Group A HR (bpm)	Group B HR (bpm)	Group A S_P_O_2_(%)	Group B S_P_O_2_(%)
Preoperative	73.67 ± 7.14	73.77 ± 7.44	97.64 ± 1.22	97.83 ± 1.40
postoperative	74.39 ± 8.74	73.94 ± 8.91	97.85 ± 1.03	97.94 ± 1.28
Pre-postoperative (F/*P* value)	0.12/0.73		0.65/0.42	
Between groups (F/*P* value)	0.11/0.74		0.26/0.61	
Pre-postoperative* Between groups (F/*P* value)	0.05/0.82		0.03/0.86	

**Table 3:** Comparison of peripheral perfusion index (PI) before and after operation between the two groups.

	Group A PI-Left	Group B PI-Right	Group A PI-Right	Group B PI-Right
Preoperative	1.71 ± 1.24	1.41 ± 1.24	2.02 ± 1.24	1.55 ± 1.05
postoperative	6.49 ± 2.61	5.92 ± 2.00	7.13 ± 3.12	7.40 ± 3.80
Pre-postoperative (F/*P* value)	333.77/*P *< 0.01		196.71/*P *< 0.01	
Between groups (F/*P* value)	1.30/0.26		0.034/0.85	
Pre-postoperative* Between groups (F/*P* value)	0.24/0.63		0.91/0.35	

**Table 4:** Comparison of pre and postoperative palm temperature (T, °C) between the two groups.

	Group A T-Left	Group B T-Left	Group A T-Right	Group B T-Right
Preoperative	31.45 ± 1.50	31.43 ± 2.14	31.47 ± 1.69	31.27 ± 2.23
postoperative	35.17 ± 1.11	34.81 ± 1.17	35.14 ± 0.94	34.39 ± 1.76
Pre-postoperative (F/*P* value)	295.70/*P *< 0.01		245.32/*P *< 0.01	
Between groups (F/*P* value)	0.38/0.54		1.75/0.19	
Pre-postoperative* Between groups (F/*P* value)	0.71/0.40		1.59/0.21	

### 3.3. Comparison of follow-up indicators

During follow-up, one patient in group A was lost at 12 months, and one patient in group B was lost at 24 months after the operation. All other patients completed the follow-up for 36 months.

The recurrence rates of the two groups were compared at 1 and 3 months after the operation (5.88% vs. 2.86%, 20.59% vs. 5.71%), with no significant difference (*P* > 0.05). However, at 6, 12, 24, and 36 months (32.35% vs. 11.43%, 32.35% vs. 11.43%, 56.25% vs. 14.71%, 68.75% vs. 20.59%), there was a statistically significant difference (*P* < 0.05) (Table 5). The difference in intercostal neuralgia between the two groups (52.94% vs. 22.86%) was also statistically significant (*P* < 0.05). All patients with intercostal neuralgia had a pain score of NRS < 4, which had no significant impact on their lives, and they all recovered within 3 months of the operation. The incidence of postoperative compensatory sweating was 19 cases (55.88%) in group A and 8 cases (22.86%) in group B, which was statistically significant (*P* > 0.05). However, all cases of compensatory hyperhidrosis in the two groups were mild to moderate, and no severe compensatory sweating occurred (Table 6).

**Table 5:** Comparison of the postoperative recurrence rate between the two groups.

Groups	1M (*n*/%)	3M (*n*/%)	6M (*n*/%)	12M (*n*/%)	24M (*n*/%)	36M (*n*/%)
A (*n* = 34)	2/5.88	7/20.59	11/32.35	11/33.33	18/56.25	22/68.75
B (*n* = 35)	1/2.86	2/5.71	4/11.43	4/11.43	5/14.71	7/20.59
*x*^2^ value	0.001	2.180	4.438	4.740	12.531	15.523
*P* value	0.980	0.140	0.035	0.029	<0.001	<0.001

**Table 6:** Comparison of the incidence of intercostal neuralgia and compensatory hyperhidrosis between the two groups.

Groups	Intercostal neuralgia	Compensatory hyperhidrosis
	Cases/percentage (*n*/%)	Cases/percentage (*n*/%)
A	18/52.94	19/55.88
B	8/22.86	8/22.86
*x*^2^ value	6.647	7.897
*P* value	0.010	0.005

## 4. Discussion

**Figure 6:**
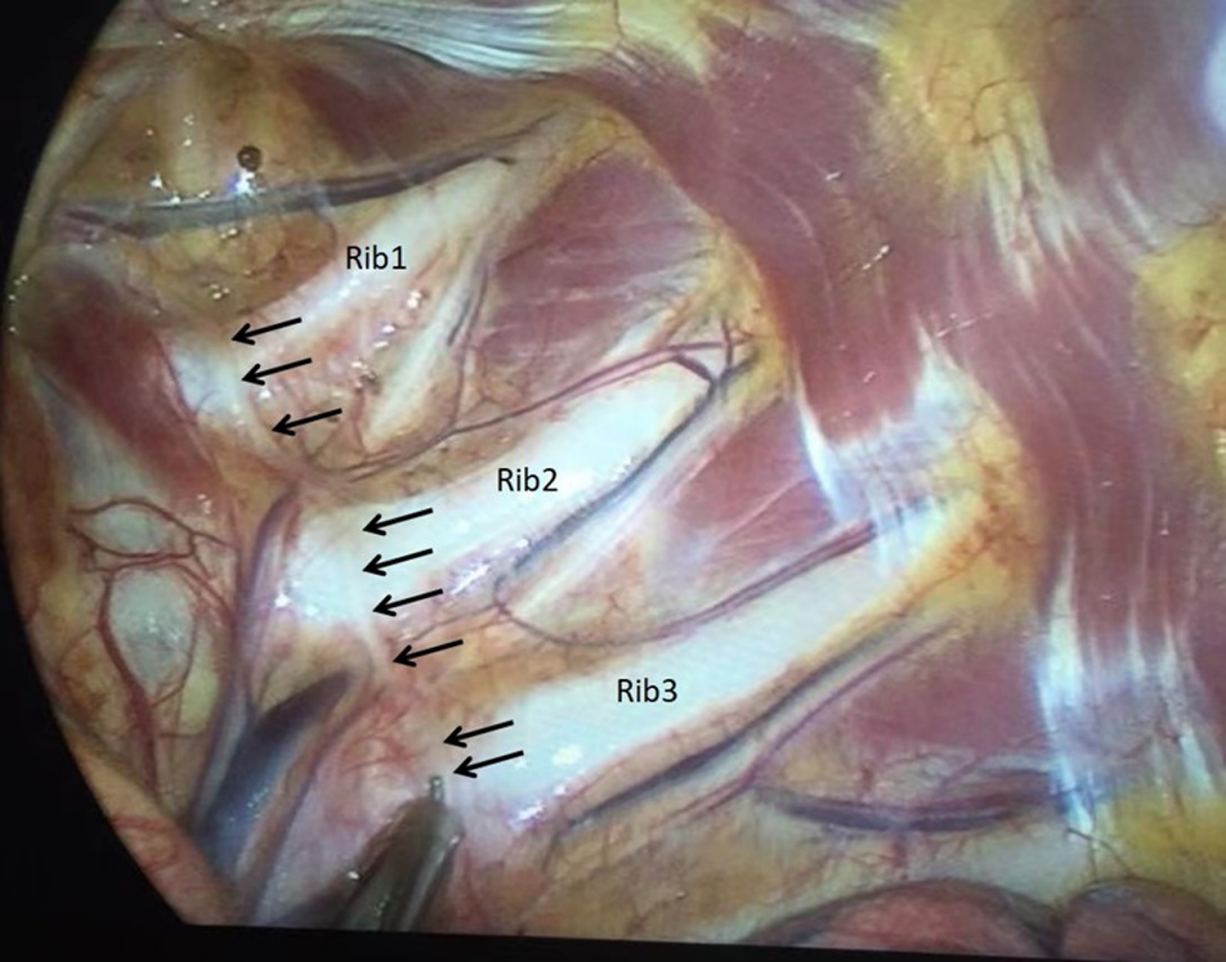
The thoracic sympathetic nerve chain is observed to cling to the pleura in front of the small head of ribs (indicated by the black arrow), rather than in the paraspinal space.

Currently, there are few effective treatments for PPH. The commonly used drugs include oral oxybutynin (15), local botulinum toxin injection (16, 17), local iontophoresis (18), local microneedle radiofrequency (19, 20), or other conservative treatments, but they have some limitations. Although ETS has high efficacy and long-term curative effects in the treatment of PPH and was once considered the “gold standard” treatment (4, 5, 21), its use is limited due to the high incidence of compensatory hyperhidrosis, which can be as high as 78% (5, 22). Compensatory hyperhidrosis is a significant problem for both doctors and patients following ETS since there is currently no effective treatment. Although some scholars claim that comprehensive bilateral thoracic sympathetic nerve chain resection performed in one sitting may be efficacious, the long-term impact of such extensive resection on human physiology remains unknown. Moreover, ETS requires general anesthesia and alternate single lung collapse, which can result in complications such as atelectasis, pneumothorax, and other postoperative complications. Under the funding of the Zhejiang Medical and Health Platform's key project, we conducted research on the treatment technology of CT-guided absolute alcohol chemical destructive block of the thoracic sympathetic nerve chain to explore a new treatment technology for palmar hyperhidrosis (6–8). This technique has been effectively utilized for managing palmar hyperhidrosis, cephalic hyperhidrosis, axillary hyperhidrosis, perineal hyperhidrosis of the lower limbs, and Raynaud's syndrome (6–9, 23, 24), as well as a combined thoracic and lumbar sympathetic nerve block for palmar and foot hyperhidrosis (25, 26). Resen et al. conducted a study on the effectiveness of CT-guided chemical destruction of the thoracic sympathetic nerve chain with absolute alcohol in treating palmar hyperhidrosis. The study included 86 cases and the results showed that the short-term curative effect was observed to be 100% (27), and the curative effect is comparable to ETS within one year but with fewer complications (28). However, although absolute alcohol chemical damage block has high immediate efficiency, the time effect is far less lasting than ETS, with a 3 year recurrence rate as high as 68.75% and a 55.88% incidence of postoperative compensatory hyperhidrosis. The liquid medication exhibits limited controllability and can easily spread along the pleura, leading to alcoholic aseptic inflammation of the affected intercostal nerve. This may result in intercostal nerve pain that can last for a duration of 1–3 months. Horner's syndrome (14) can also occur if it flows upward to the level of the pleura crest. If the injected absolute alcohol accidentally enters the subarachnoid space or causes a spinal root artery embolism, it can cause serious complications such as paraplegia (29). With the support of Jiaxing's special funds for important scientific research, our focus was redirected toward radiofrequency thermocoagulation and its corresponding physical damage to the sympathetic nerve chain (9). In a meta-analysis conducted by Hasimoto et al. (30), nine studies on the radiofrequency treatment of palmar hyperhidrosis with the thoracic sympathetic nerve chain were analyzed. The results revealed that radiofrequency thermocoagulation therapy was found to have a short-term curative effect that was comparable to ETS. Although the long-term recurrence rate is slightly higher than that of ETS, the incidence of compensatory hyperhidrosis of radiofrequency therapy is significantly lower than that of ETS. According to Andrade et al., percutaneous radiofrequency ablation of the thoracic sympathetic nerve chain is a potentially feasible and safe method to treat palmar hyperhidrosis (31). Using relatively rough C-arm x-ray guidance to perform radiofrequency thermocoagulation of the thoracic sympathetic nerve chain to treat palmar hyperhidrosis has a better curative effect than local carnitine injection, as stated by Mostafa et al. (31). In this study, our results showed that both the immediate effectiveness rate of absolute alcohol damage block of the thoracic sympathetic chain and radiofrequency can achieve a 100% success rate. There was no significant difference in the recurrence rate between the two groups at 1 month and 3 months after operation (*P* > 0.05). In contrast, the long-term recurrence rate of the latter is markedly lower compared with that of the former. The incidence of intercostal neuralgia and hypobaric hyperhidrosis after surgery was significantly lower than that of the former. The reason for the regenerative and resurrective properties of the sympathetic nerve chain may be attributed to the relatively low cytotoxicity of absolute alcohol towards the nerve tissue. Palmar hyperhidrosis was difficult to recur after radiofrequency because the nerve chain was completely physically damaged and lost its ability to regenerate and revive. According to Garca-Barqun et al.,139 cases of palmar hyperhidrosis were treated with bilateral T2, T3, and T4 thoracic sympathetic nerve chains at 90°C for 8 min (32). The remission rate after one month was only 77.38%, significantly lower than the remission rate after radiofrequency treatment of only one segment (T4) in this group. Following meticulous examination of the described method, it was ascertained that the radiofrequency needle failed to accurately target the anterior superior edge of the small head of the ribs. Instead, it inadvertently penetrated the paravertebral space situated external to the sympathetic nerve chain within the body (Figure 6). Therefore, even more, RF time and RF segments are insufficient to achieve the desired effect. The therapeutic target is located at the anterior superior edge of the capitulum in the fourth rib, and precise adherence of the radiofrequency puncture needle to the parietal pleura is necessary to avoid the potential risk of pneumothorax resulting from accidental pleural puncture. To address this concern, we developed and manufactured a unique puncture needle for thoracic sympathetic nerve radiofrequency. The needle has a depth scale on the needle body and a blunt tip, reducing the occurrence of pneumothorax complications (33).

## 5. Conclusion

In conclusion, our study suggests that CT-guided percutaneous thoracic sympathetic nerve chain nerve block with absolute alcohol and radiofrequency thermocoagulation are both effective treatments for PPH. However, radiofrequency thermocoagulation appears to be more durable, with a lower recurrence rate and fewer complications such as intercostal neuralgia and compensatory sweating. These findings highlight the potential of radiofrequency thermocoagulation as a safe and effective treatment option for PPH.

## Data Availability

The raw data supporting the conclusions of this article will be made available by the authors, without undue reservation.
